# Supplementary material for: Bogolon-mediated light absorption in atomic condensates of different dimensionality
Source: Sci Rep. 2023 Apr 19;13:6358. doi: 10.1038/s41598-023-33091-5 (PMC10115858; doi:10.1038/s41598-023-33091-5)
Supplement: Supplementary file 1 — Supplementary Information. [file 41598_2023_33091_MOESM1_ESM.pdf]

Supplemental material

Dogyun Ko[1,2], Meng Sun[3,1], Vadim Kovalev[4,5], Ivan Savenko[1,2]

[1] Center for Theoretical Physics of Complex Systems, Institute for Basic Science (IBS), Daejeon 34126, Korea
[2]Basic Science Program, Korea University of Science and Technology (UST), Daejeon 34113, Korea
[3] Faculty of Science, Beijing University of Technology, Beijing 100124, China
[4]A.V. Rzhanov Institute of Semiconductor Physics, Siberian Branch of Russian Academy of Sciences, Novosibirsk 630090, Russia
[5]Novosibirsk State Technical University, Novosibirsk 630073, Russia

This supplemental material provides the details of evaluation of all the integrals for the absorption probabilities in various dimensionalities. First, to find $I^1_{2b,\mathrm{2D}}$ we set $q = |\mathbf{p} + \mathbf{k}_{||}|$. Thus, the in-plain angle θ between momenta $\mathbf{p}$ and $\mathbf{k}_{||}$ reads

$$\sin\theta = \frac{\sqrt{[(p+k_{||})^2 - q^2][q^2 - (p-k_{||})^2]}}{2pk_{||}}, \tag{1}$$

and

$$d\theta = -qdq/(pk_{||}\sin\theta) = -2\frac{qdq}{\sqrt{[(p+k_{||})^2 - q^2][q^2 - (p-k_{||})^2]}}. \tag{2}$$

Substitution Eq. (2) in $I^1_{2b,\mathrm{2D}}$ yields

$$\begin{aligned} I^1_{2b,\mathrm{2D}} &= \int\limits_0^\infty pdp \int\limits_0^{2\pi} d\theta \frac{1}{p|\mathbf{p}+\mathbf{k}_{||}|}\delta(|\mathbf{p}+\mathbf{k}_{||}| + p - \omega_\mathbf{k}/s) \\ &= 4\int\limits_0^\infty dp \int\limits_{|p-k_{||}|}^{|p+k_{||}|} dq \frac{\delta\left(q + p - \omega_\mathbf{k}/s\right)}{\sqrt{[(p+k_{||})^2 - q^2][q^2 - (p-k_{||})^2]}}. \end{aligned} \tag{3}$$

Furthermore, we employ the Lagrange multipliers by the change of variables, $q + p = x$ and $q - p = y$, thus

$$\int\limits_0^\infty dp \int\limits_{|p-k_{||}|}^{|p+k_{||}|} dq = \int\limits_{k_{||}}^\infty dx \int\limits_{-k_{||}}^{k_{||}} dy \left|\frac{\partial(p,q)}{\partial(x,y)}\right|. \tag{4}$$

Substituting Eq. (4) in Eq. (3) gives

$$\begin{aligned} I^1_{2b,\mathrm{2D}} &= 2\int\limits_k^\infty dx \int\limits_{-k_{||}}^{k_{||}} dy \frac{\delta\left(x - \omega_\mathbf{k}/s\right)}{\sqrt{(x^2 - k_{||}^2)(k_{||}^2 - y^2)}} \\ &= 2\pi\int\limits_{k_{||}}^\infty dx \frac{\delta\left(x - \omega_\mathbf{k}/s\right)}{\sqrt{x^2 - k_{||}^2}} = \frac{2\pi\Theta(\omega_\mathbf{k}/s - k_{||})}{\sqrt{(\omega_\mathbf{k}/s)^2 - k_{||}^2}}. \end{aligned} \tag{5}$$

A similar method can be employed to calculate $I^1_{2b,3\mathrm{D}}$,

$$I^1_{2b,3\mathrm{D}} = 2\pi \int\limits_0^\infty p^2 dp \int\limits_{|p+k|}^{|p-k|} \frac{qdq}{pk} \frac{1}{pq} \delta\left(q + p - \omega_{\mathbf{k}}/s\right)$$

$$= \frac{\pi}{k} \int\limits_k^\infty dx \int\limits_{-k}^{k} dy \delta\left(x - \omega_{\mathbf{k}}/s\right) = 2\pi\Theta(\omega_{\mathbf{k}}/s - k). \tag{6}$$

Next, for 1b processes

$$I^3_{1b,1\mathrm{D}} = \int d\mathbf{p} \frac{1}{|p|} \delta\left(\frac{\Delta_\eta}{s} + p - \frac{\omega_{\mathbf{k}}}{s}\right) = \int\limits_{-\infty}^{\infty} \frac{dp}{|p|} \delta\left(\frac{\Delta_\eta}{s} + p - \frac{\omega_{\mathbf{k}}}{s}\right) = 2s \frac{\Theta[\omega_{\mathbf{k}} - \Delta_\eta]}{\omega_{\mathbf{k}} - \Delta_\eta}, \tag{7}$$

in the case of 1D BEC. The corresponding expression for 2D condensate reads

$$I^3_{1b,2\mathrm{D}} = \int d\mathbf{p} \frac{1}{p} \delta\left(\frac{\Delta_\eta}{s} + p - \frac{\omega_{\mathbf{k}}}{s}\right) = \int\limits_0^\infty dp \int\limits_0^{2\pi} d\theta \delta\left(\frac{\Delta_\eta}{s} + p - \frac{\omega_{\mathbf{k}}}{s}\right) \tag{8}$$

$$= 2\pi \int\limits_0^\infty dp \delta\left(\frac{\Delta_\eta}{s} + p - \frac{\omega_{\mathbf{k}}}{s}\right) = 2\pi\Theta[\omega_{\mathbf{k}} - \Delta_\eta],$$

and, finally, for 3D BEC one finds

$$I^3_{1b,3\mathrm{D}} = \int d\mathbf{p} \frac{1}{p} \delta\left(\frac{\Delta_\eta}{s} + p - \frac{\omega_{\mathbf{k}}}{s}\right) = \int\limits_0^\infty pdp \int\limits_0^{\pi} \sin\theta d\theta \int\limits_0^{2\pi} d\phi \delta\left(\frac{\Delta_\eta}{s} + p - \frac{\omega_{\mathbf{k}}}{s}\right) \tag{9}$$

$$= 4\pi \int\limits_0^\infty pdp \delta\left(\frac{\Delta_\eta}{s} + p - \frac{\omega_{\mathbf{k}}}{s}\right) = \frac{4\pi}{s}(\omega_{\mathbf{k}} - \Delta_\eta)\Theta[\omega_{\mathbf{k}} - \Delta_\eta].$$

These expressions are analyzed in the main text.
